# Idiopathic Concurrent Pulmonary Embolism and Pulmonary Vein Thrombosis: A Rare Entity

**DOI:** 10.7759/cureus.78584

**Published:** 2025-02-05

**Authors:** Tousif Baig, Sugeesha Wickramasinghe

**Affiliations:** 1 Respiratory Medicine, Lancashire Teaching Hospitals NHS Foundation Trust, Preston, GBR

**Keywords:** pulmonary embolism, pulmonary hypertension, pulmonary vein thrombosis, rare lung disease, right heart strain

## Abstract

The concurrent presence of pulmonary embolism (PE) and pulmonary vein thrombosis (PVT) is an extremely rare entity, as there is not much evidence in the literature. PVT is frequently associated with lung transplants or surgery.

We describe two cases of idiopathic concurrent PE and PVT with a literature review to highlight the challenges of management and evaluation of these cases. The first case describes a young female who presented with lower limb venous thrombosis with a subsequent course of PE and PVT. Despite extensive investigations, no underlying cause was identified. The second case involves a middle-aged female with recurrent PE and a new PVT. Both patients were started on long-term anticoagulation therapy due to the recurrent nature and severity of their disease.

This case series highlights the challenges associated with the management and evaluation of this rare clinical entity. Further research is needed for a better understanding of the underlying mechanisms and optimal treatment.

## Introduction

The concurrent presence of pulmonary embolism (PE) and pulmonary venous thrombosis (PVT) is an extremely rare entity, presenting a treatment challenge due to limited evidence in the literature.

PVT is frequently associated with primary lung malignancies, lung transplant, post-lung surgery, or atrial fibrillation [1]. However, idiopathic combined PE and PVT is an extremely rare entity and, to our knowledge, has not been previously described before in the literature.

Here, we describe two cases of idiopathic PE and PVT, along with a literature review to highlight the management challenges of this combination. 

## Case presentation

Case series 

*Case 1* 

A 21-year-old previously healthy female presented with left leg pain, numbness, swelling, and left iliac fossa pain. She had no significant medical comorbidities. Her mother was diagnosed with antiphospholipid antibody syndrome and she had a family history of Factor V Leiden deficiency.

An ultrasound venous Doppler revealed thrombosis of the left popliteal vein. Initial treatment with therapeutic-dose enoxaparin and leg elevation failed to resolve the deep vein thrombosis symptoms, necessitating interventional radiology-guided venous thrombectomy and high-pressure venoplasty. Despite the initial successful procedure, a follow-up Doppler revealed that the popliteal vein had re-thrombosed. 

**Figure 1:**
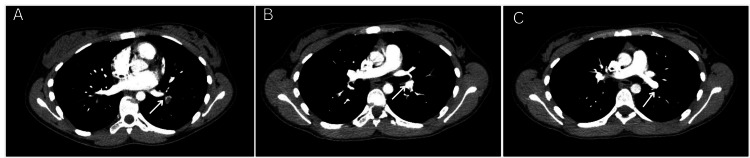
Axial CT pulmonary angiography (CTPA) images (A, B, and C) from Case 1 demonstrating multiple filling defects within proximal segmental pulmonary artery branches supplying the left lower lung lobe (arrows)

**Figure 2:**
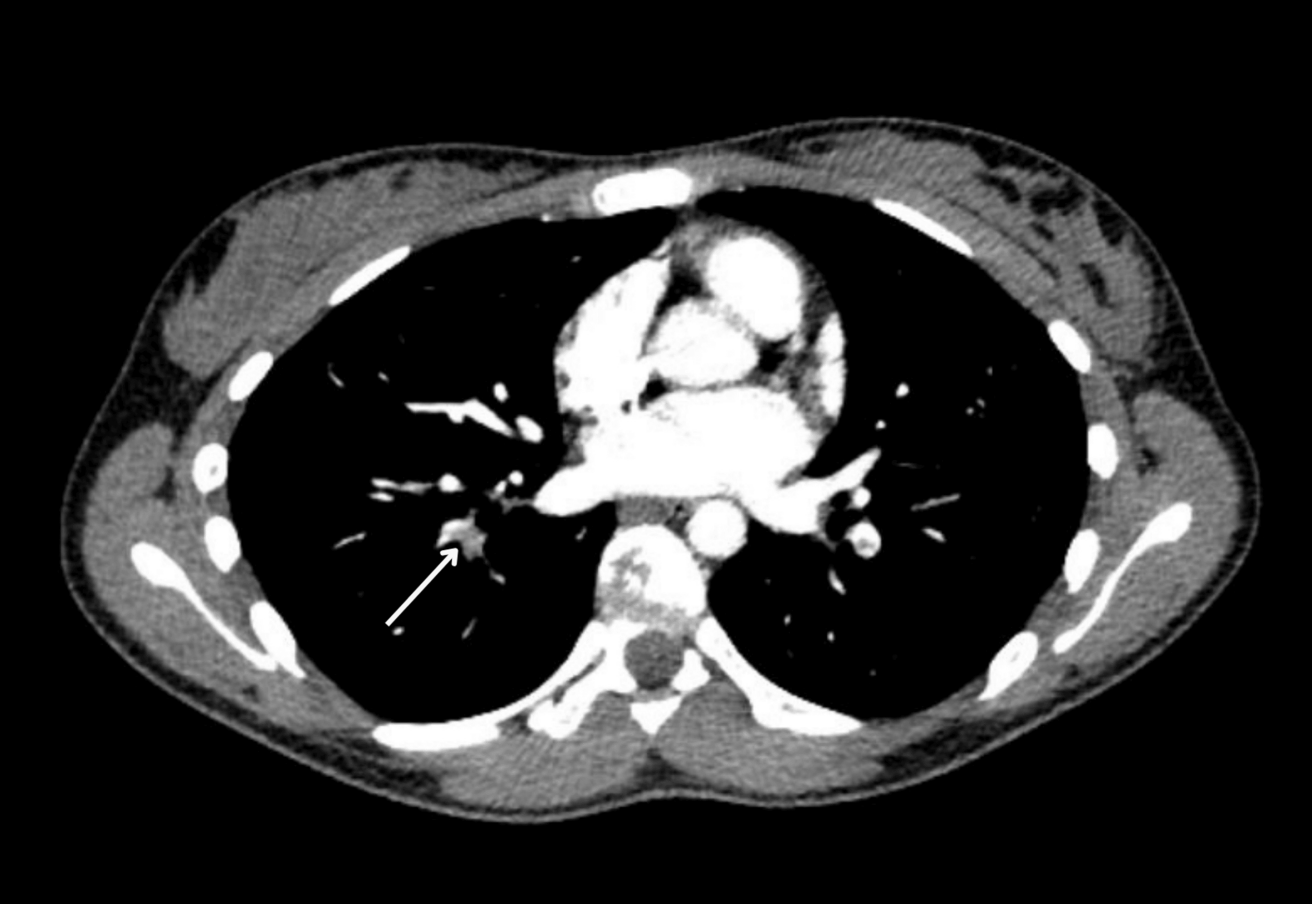
Axial CT pulmonary angiography (CTPA) image from Case 1 demonstrating a filling defect within a segmental pulmonary artery branch supplying the right lower lung lobe (arrow)

Two weeks after the initial presentation, she experienced pleuritic chest pain and shortness of breath. Further evaluation with a CT pulmonary angiogram showed extensive bilateral pulmonary artery embolism (Figures 1, 2).

**Figure 3:**
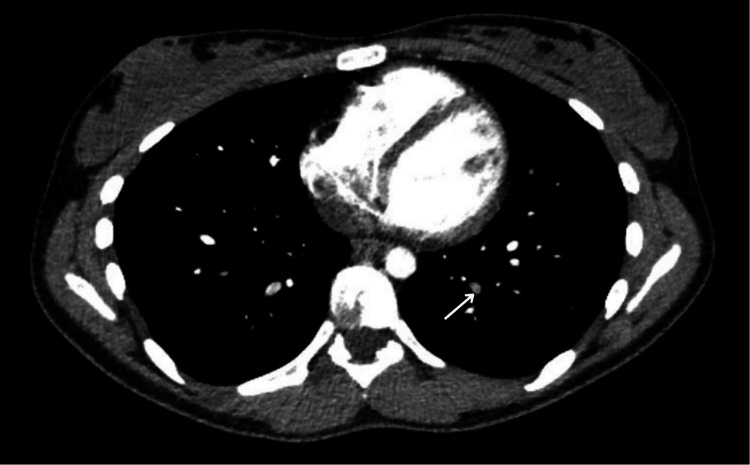
CT pulmonary angiography (CTPA) axial image demonstrating a filling defect within the segmental pulmonary vein branch draining the lower lobe of the left lung (arrow)

In addition to the extensive bilateral PE, the CT pulmonary angiography (CTPA) also revealed thrombosis of the pulmonary vein branch draining the lower lobe of the left lung (Figure 3). 

While in the hospital, she was treated briefly with treatment-dose subcutaneous enoxaparin; she improved symptomatically after treatment with enoxaparin. She was discharged with oral anticoagulation (apixaban) with further follow-up arranged in the Specialist Pulmonary Embolism Clinic. A detailed evaluation was conducted in the Specialist Pulmonary Embolism Clinic. A comprehensive history did not reveal any obvious provoking factors.

She was referred to haematology for further evaluation. A panel of investigations was performed to evaluate for antiphospholipid antibodies, Factor V Leiden mutation, and thrombophilias, which were all negative. Further haematological testing for JAK2 mutation and paroxysmal nocturnal haemoglobinuria (PNH) was negative. She was also investigated with a bubble contrast to rule out intra-cardiac shunts.

The patient had been started on long-term anticoagulation with apixaban due to the extensive and unprovoked nature of the thromboembolic disease. Three months after the initial presentation, an echocardiogram was done which did not show any evidence of pulmonary hypertension. She is currently under active follow-up with the Specialist Outpatient Pulmonary Embolism Service.

*Case 2* 

A 45-year-old female was referred to the respiratory clinic for further evaluation of pulmonary thromboembolism. She was admitted two years ago with pneumonia and parapneumonic effusion. A CTPA was done at that time due to ongoing oxygen requirement despite adequate treatment for pneumonia. The CTPA showed an embolism of the pulmonary artery supplying the right lower lung lobe, and this was considered provoked due to concurrent pneumonia.

She was treated with a course of antibiotics for pneumonia and was discharged with a three-month course of oral anticoagulation (apixaban) for the provoked PE.

The patient was followed up in the respiratory clinic, having remained symptomatically well, and her parapneumonic effusion had resolved; she was subsequently discharged from the respiratory clinic.

Eighteen months after the initial episode of pneumonia and PE, she presented again with acute-onset pleuritic-type chest pain, breathlessness, and haemoptysis. A detailed history at this point did not identify any provoking factors for PE.

**Figure 4:**
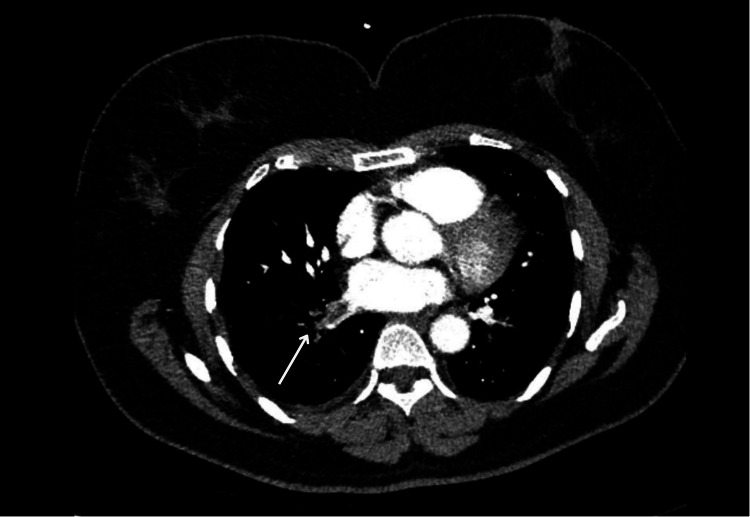
Axial CT pulmonary angiography (CTPA) images from Case 2 demonstrating a large filling defect within the right inferior pulmonary vein extending from segmental and subsegmental branches of the right lower lung lobe (arrow)

**Figure 5:**
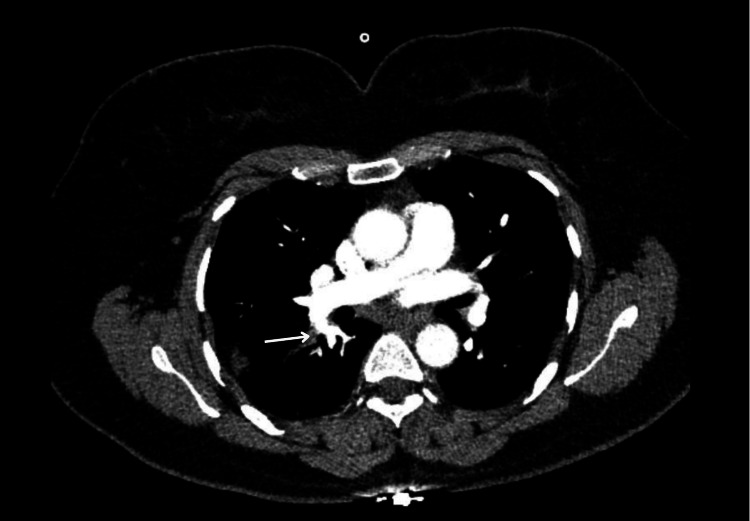
Axial CT pulmonary angiography (CTPA) image from Case 2 demonstrating a filling defect in the segmental pulmonary artery branch supplying the superior part of the right lower lung lobe (arrow)

**Figure 6:**
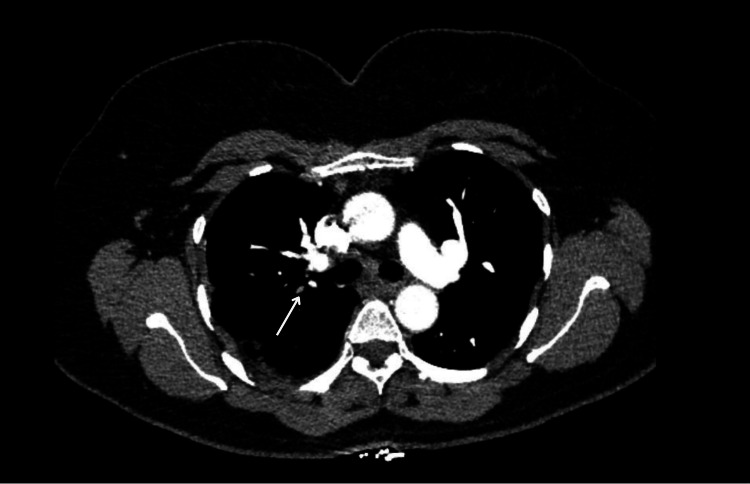
Axial CT pulmonary angiography (CTPA) image from Case 2 demonstrating a small filling defect within the segmental venous branches of the right upper lung lobe (arrow)

A CTPA done at that time revealed significant thrombosis of the right inferior pulmonary vein extending from segmental and subsegmental branches of the right lower lung lobe (Figure 4), embolism of the pulmonary artery branch supplying the lower lobe of the left lung (Figure 5), and a small thrombus in the segmental pulmonary venous branches draining the right upper lung lobe (Figure 6).

The patient was started on apixaban and as she remained symptomatically well and haemodynamically stable; she was discharged and a follow-up was arranged in the Specialist Pulmonary Embolism Clinic. 

The patient was evaluated in the Specialist Pulmonary Embolism Clinic where a comprehensive history did not identify any provoking factors for thromboembolism. She was also referred to haematology for further evaluation. All investigations including thrombophilia screen, antiphospholipid antibody screen, PNH panel, and connective tissue disease screening were negative.

Echocardiogram was done which showed normal biventricular function without any evidence of pulmonary hypertension. Bubble contrast of the heart did not show intra-cardiac shunts.

She was started on long-term oral anticoagulation (apixaban) due to the recurrent and extensive nature of the thromboembolic disease. Three months after initiating anticoagulation, an echocardiogram was performed, which did not show any evidence of pulmonary hypertension. She is currently under active follow-up with the Specialist Outpatient Pulmonary Embolism service. 

## Discussion

PVT is a rare condition that is mostly associated with major pulmonary surgery and pulmonary malignancies [1]. In addition, this is described following radiofrequency catheter ablation for atrial fibrillation [2]. In addition, it has been described in association with trauma, arteriovenous malformations, and atrial myxoma [1]. Patients present with nonspecific symptoms such as chest pain, breathlessness, cough, and haemoptysis and these can mimic PE. PVT can lead to an increase in pressure in pulmonary veins which can cause compensatory pulmonary arterial vasoconstriction leading to pulmonary hypertension and cor pulmonale. Complications such as stroke or transient ischaemic attacks due to peripheral embolization, pulmonary gangrene and secondary bacterial infections in the affected segments, and lung fibrosis were described [3,4]. 

Pulmonary arterial thrombosis is more frequent compared to PVT but up to 33-50% will be idiopathic despite thorough evaluation. Most cases follow a benign course, with only 2-5% developing chronic thromboembolism which can get complicated with pulmonary hypertension. 

Concurrent pulmonary arterial and pulmonary venous thrombosis is an extremely rare entity. These patients should be thoroughly investigated for underlying causes such as thrombophilia, malignancies, and connective tissue disorders, which were negative in our cases. Patients underwent bubble contrast echocardiogram to rule out septal defects that may cause shunts, allowing passage of blood clots from left atrium to right atrium, causing embolization to pulmonary arteries.

Patients have also been evaluated for other conditions that can cause arterial and venous thrombosis such as anti-phospholipid syndrome and paroxysmal haemoglobinuria which were negative. 

Both of our patients were relatively young, and we could not identify any underlying etiology. Based on the clot burden and the severity of thrombosis, they were started on long-term anticoagulation after assessing the risks of bleeding and the benefits of anticoagulation. Additionally, it was decided to monitor them for the development of pulmonary hypertension. 

Certain case reports describe the presence of PVT with COVID-19 infection which may be related to the pro-thrombotic nature of COVID-19, which is well described in the literature [5]. However, the involvement of the pulmonary venous circulation is an area that needs evaluation as there is no evidence or studies available to explain this. 

Ohtaka et al. reported 18 patients with PVT following left upper lobectomy (LUL) [6], speculating that the cause of thrombosis in the left superior pulmonary vein (LSPV) stump after LUL was a long LSPV stump. It might develop because turbulent flow or stasis of blood occurs in the long PV stump [6]. Furthermore, Kwek et al. reported that thrombosis developed in longer PV stumps [6]. In a short PV stump, blood flow may occur because blood flow in the left atrium spreads through the entire PV stump. 

Treatment of PVT should be determined on the basis of the obstructing pathological finding and can include antibiotic therapy, anticoagulation, thrombectomy, and/or pulmonary resection [7]. Thrombectomy can been successfully performed when medical therapy fails. Lobectomy may be indicated when PVT is complicated with massive haemoptysis or pulmonary necrosis when other measures fail [8]. 

Despite aetiology, there is no evidence to determine the preferred duration of anticoagulation or preference for the modality of anticoagulation between oral vitamin K antagonists, direct oral anticoagulants, or heparin. Further studies are needed to address this issue but this is extremely limited given the rare occurrence of the combined thrombosis in the pulmonary vasculature. 

## Conclusions

This case series presents two unique instances of concurrent PE and PVT, a rare and challenging clinical entity. Despite extensive investigations, no definitive underlying etiology could be identified in either patient.

Given the severity of the condition and the risk of complications like pulmonary hypertension, both patients were initiated on long-term anticoagulation therapy. The optimal duration and modality of anticoagulation in such cases remain uncertain, as evidence-based guidelines are lacking.
